# Ultra-fast scalable estimation of single-cell differentiation potency from scRNA-Seq data

**DOI:** 10.1093/bioinformatics/btaa987

**Published:** 2020-11-27

**Authors:** Andrew E Teschendorff, Alok K Maity, Xue Hu, Chen Weiyan, Matthias Lechner

**Affiliations:** CAS Key Lab of Computational Biology, CAS-MPG Partner Institute for Computational Biology, Shanghai Institute of Nutrition and Health, Shanghai Institutes for Biological Sciences, University of Chinese Academy of Sciences, Chinese Academy of Sciences, Shanghai 200031, China; UCL Cancer Institute, University College London, London WC1E 6BT, UK; CAS Key Lab of Computational Biology, CAS-MPG Partner Institute for Computational Biology, Shanghai Institute of Nutrition and Health, Shanghai Institutes for Biological Sciences, University of Chinese Academy of Sciences, Chinese Academy of Sciences, Shanghai 200031, China; CAS Key Lab of Computational Biology, CAS-MPG Partner Institute for Computational Biology, Shanghai Institute of Nutrition and Health, Shanghai Institutes for Biological Sciences, University of Chinese Academy of Sciences, Chinese Academy of Sciences, Shanghai 200031, China; CAS Key Lab of Computational Biology, CAS-MPG Partner Institute for Computational Biology, Shanghai Institute of Nutrition and Health, Shanghai Institutes for Biological Sciences, University of Chinese Academy of Sciences, Chinese Academy of Sciences, Shanghai 200031, China; UCL Cancer Institute, University College London, London WC1E 6BT, UK; Department of Otolaryngology-Head and Neck Surgery, Stanford University School of Medicine, Palo Alto, CA 94305-5739, USA

## Abstract

**Motivation:**

An important task in the analysis of single-cell RNA-Seq data is the estimation of differentiation potency, as this can help identify stem-or-multipotent cells in non-temporal studies or in tissues where differentiation hierarchies are not well established. A key challenge in the estimation of single-cell potency is the need for a fast and accurate algorithm, scalable to large scRNA-Seq studies profiling millions of cells.

**Results:**

Here, we present a single-cell potency measure, called Correlation of Connectome and Transcriptome (CCAT), which can return accurate single-cell potency estimates of a million cells in minutes, a 100-fold improvement over current state-of-the-art methods. We benchmark CCAT against 8 other single-cell potency models and across 28 scRNA-Seq studies, encompassing over 2 million cells, demonstrating comparable accuracy than the current state-of-the-art, at a significantly reduced computational cost, and with increased robustness to dropouts.

**Availability and implementation:**

*CCAT* is part of the SCENT R-package, freely available from https://github.com/aet21/SCENT.

**Supplementary information:**

Supplementary data are available at *Bioinformatics* online.

## 1 Introduction

One of the key aims of single-cell RNA-Seq studies is to elucidate the differentiation hierarchies of complex tissues (Rozenblatt-Rosen et al., 2017). This requires the unbiased estimation of differentiation potency of single-cells, to enable identification of stem-or-multipotent progenitor cells, and ranking of single cells along differentiation potency gradients (Teschendorff and Enver, 2017). These tasks are particularly challenging in non-temporal scRNA-Seq studies, or in tissues where differentiation hierarchies are not well established (Chen et al., 2019). Although tools such as pseudotime (Trapnell et al., 2014) can be adapted to non-temporal scRNA-Seq data, it requires a priori identification of root-states, often through use of prior biological knowledge (e.g. surface marker expression), which can be highly ambiguous and biased. Because of this, recent methods (Banerji et al., 2013; Guo et al., 2017; Teschendorff and Enver, 2017) have aimed to estimate differentiation potency of single-cells from the ‘bottom-up’, that is, by using only information from a cell’s network state, without the need for prior biological knowledge. In principle, such bottom-up approaches provide improved and less biased estimates of differentiation potency. A recent comparative study (Gulati et al., 2020) of such single-cell potency measures identified CytoTRACE (Gulati *et al.*, 2020) and SCENT (Teschendorff and Enver, 2017) as two of the best performing methods. However, a numerical challenge remains in that the estimation of single-cell potency with methods like CytoTRACE and SCENT can be computationally intensive, requiring runtimes of many hours, days or even weeks, depending on cell number and available computational resources.

Here, we present an ultra-fast method, called CCAT, for estimating differentiation potency of single cells from scRNA-Seq data, which is scalable to upcoming scRNA-Seq studies profiling millions of cells. Under certain assumptions, CCAT can be viewed as providing a fast proxy to our previously proposed Single-Cell Entropy (SCENT) (also known as entropy-rate *SR*) measure (Teschendorff and Enver, 2017), but we stress that CCAT represents a different method which is not mathematically equivalent to SCENT. We evaluate and benchmark CCAT against 8 other single-cell potency measures across 28 scRNA-Seq studies encompassing over 2.1 million cells, demonstrating that CCAT achieves comparable performance to state-of-the-art methods like CytoTRACE or SCENT, with an approximate 10–1000-fold improvement in computational efficiency. We demonstrate CCAT’s accuracy and scalability on a scRNA-Seq study profiling two million cells, its robustness to noise, and show how it can be integrated with existing linage trajectory inference algorithms to provide unambiguous assignment of root-states or identification of stem-like states.

## 2 Materials and methods

### 2.1 Estimating differentiation potency with CCAT

The CCAT measure is directly motivated by our previously proposed entropy-rate (*SR*) (Banerji *et al.*, 2013; Teschendorff and Enver, 2017). Given an appropriately normalized scRNA-Seq profile $x=(x_{1},\ldots ,x_{G})$ where *G* is the number of genes overlapping with a protein-protein-interaction (PPI)-network, so that the *G *×* G* adjacency matrix *A* is connected, we first define a stochastic diffusion matrix *P* on this graph, by the entries

(1) $$p_{ij}=\frac{A_{ij}x_{j}}{\sum_{k}A_{ik}x_{k}}.$$

In the above, *A_ii_* = 0 and *A_ij_* = 0 if *i* and *j* are not neighbors in the network, with *A_ij_* = 1 if and only if *i* and *j* are neighbors. From the stochastic matrix, the entropy-rate (*SR*) is defined by

(2) $$SR=-\sum_{i,j}\pi_{i}p_{ij}\;\text{log}\;p_{ij},$$

where *π* is the invariant measure of the Markov Chain process on the graph, satisfying πP=π and the detailed balance equation $\pi_{i}p_{ij}=\pi_{j}p_{ji}$. It can be shown that the invariant measure is given by $\pi_{i}=x_{i}{\left(Ax\right)}_{i}/x^{T}Ax$. The above equation for *SR* can also be expressed as $SR=\sum_{i}\pi_{i}S_{i}$ where the *S_i_* are the local node (gene) entropies. As explained by us previously (Teschendorff and Enver, 2017), if we take a global mean field approximation, we can replace ${\left(Ax\right)}_{i}$ up to a constant scaling with *k_i_*, the degree/connectivity of node/gene *i* in the network. Thus, *SR* can be approximated as the 3-way correlation

(3) $$SR∼\sum_{i}x_{i}k_{i}S_{i}.$$

In practice, the dynamic range of the local entropies *S_i_* is small, which means that *SR* can be further approximated proportionally by the dot product of the transcriptome **x** and connectome **k**. Indeed, we here propose the following differentiation potency measure, called CCAT (Correlation of Connectome and Transcriptome), defined by the Pearson Correlation Coefficient (PCC)

(4) CCAT=PCC(x,k)

which has the advantage of being normalized between -1 and 1. In addition, being estimated from a Pearson Correlation, it provides an ultra-fast method to estimate differentiation potency of single-cells, as there is no need to compute the invariant measure or local entropies.We stress that to derive CCAT we made a number of assumptions, and that it is therefore not mathematically equivalent to *SR*. Indeed, one can conceive of networks where the two measures are anti-correlated (Teschendorff and Enver, 2017). However, for PPI networks, CCAT provides a positively correlated proxy to *SR*.

### 2.2 Biological justification underlying CCAT

In previous studies, we have observed that high-potency cells tend to overexpress network hubs (Shi et al., 2018; Teschendorff and Enver, 2017). The overexpression of network hubs thus draws-in signaling flux, allowing signaling to be distributed more efficiently over the whole network. This explains why the entropy-rate (*SR*), which in effect measures the efficiency of a diffusion process to explore the whole network, correlates with cell potency. Thus, approximating *SR* with CCAT is sensible because CCAT directly measures the correlation between expression and node degree and therefore will be positive if the majority of network hubs are overexpressed in the more potent cells.

### 2.3 Normalization of scRNA-Seq datasets

We always use scRNA-Seq data normalized on a log-scale, in order to stabilize the variance of highly expressed genes. Because the stochastic matrix involves a ratio of gene-expression values, one needs to avoid zero values in the data matrix, so it is necessary to use a pseudocount of 1.1, so that the log(counts + 1.1) transformation takes on a minimum value above zero. Details of the scRNA-Seq datasets and the normalization procedure used in each one are available in Supplementary Information.

### 2.4 Construction of PPI network and integration with expression

In this work, we have considered a total of three different PPI networks. Two of these networks are derived from the Pathway Commons (PC) resource (Cerami et al., 2011; Rodchenkov et al., 2020), and represent two different releases (PC1 and PC2v11). The other network is derived from the STRING database v11 (Szklarczyk et al., 2019). Briefly, from PC, we downloaded the human PPI network derived from all sources, subsequently selecting interactions annotated to the following interaction types: ‘interacts-with’, ‘reacts-with’, ‘in-complex-with’, ‘used-to-produce’ and from the following databases: HPRD, BioGRID, BIND, PID, IntAct, PANTHER, KEGG, Reactome, CORUM, DIP and NetPath (Rodchenkov *et al.*, 2020). We further integrated the resulting network with protein cellular localization data from the HPRD (Prasad et al., 2009), annotating each protein to its dominant cellular domain: extracellular, cellular membrane and intra-cellular. Specifically, we removed interactions involving proteins annotated as extracellular with proteins annotated as intracellular. Finally, for the resulting network we extracted the maximally connected component. Integration with gene expression then proceeds by overlaying expression values over the nodes in the maximally connected subnetwork obtained after integrating the PPI with the genes present in the mRNA expression profile. In the case of the STRING database v11, we applied the same procedure described above to remove interactions between proteins annotated to separate intra and intercellular domains. In total, the 3 resulting PPI networks contained 8.434 nodes and 303 600 edges (PC1), 12 649 nodes and 464 091 edges (PCv12) and 12 921 nodes and 3 594 088 edges (STRING), representing 0.8%, 0.6% and 4% of the maximum possible number of edges. We note that the filtering with protein cellular localization data reduces the dimensionality of the PPI networks from around 18 000–20 000 genes to the approximately 8000 or 12 000 genes noted above.

### 2.5 Other single-cell potency estimation methods

Benchmarking of CCAT was done against eight other methods: SR/SCENT (Teschendorff and Enver, 2017), CytoTRACE (Gulati *et al.*, 2020), SLICE (Guo *et al.*, 2017), StemID (Grun et al., 2016), scEnergy (Jin et al., 2018), cmEntropy (Kannan et al., 2020) and two measures related to CytoTRACE: the number of detected genes (gene-count) and the gene-count signature (GCS) (Gulati *et al.*, 2020). Briefly, SLICE computes a Shannon entropy over a gene-ontology (GO) cluster activation profile, where the activation level of a GO-cluster is derived from the average expression of genes mapping to that GO-cluster. StemID is obtained as the Shannon entropy of a cell’s transcriptome, i.e. how uniformly distributed the read counts are among all genes. scEnergy is similar to *SR*, but instead of a PPI network, it infers a correlation network from the scRNA-Seq data itself. The GCS measure is calculated as the geometric average expression of the top-200 genes that correlate most strongly with the number of detected genes per cell, where correlations are computed for each dataset separately. The CytoTRACE measure introduces an additional smoothing step to denoise the GCS estimate. cmEntropy is similar to StemID in that it computes the Shannon entropy of read counts over genes, but focusing on the top-1000 most highly expressed genes.

### 2.6 Evaluation strategy and metric

scRNA-Seq datasets were chosen where an objective comparison between cells of high differentiation potency with cells of lower potency is possible. For instance, in developmental or differentiation timecourses, we typically compare cells of high potency at the start of the timecourse to lower potency cells at the end of the timecourse, based on the reasonable assumption that if start and end timepoints are well separated, that a potency estimation method yielding a higher discrimination accuracy between these cell groups is indicative of a better method. Specifically, we use the Area-Under-Curve (AUC) to evaluate discrimination accuracy, as derived from the statistic of a Wilcoxon rank sum test. Details of the specific cell groups being compared and the number of cells in each group for each dataset, can be found in Supplementary Information.

### 2.7 Selection of root-state and root-cell from CCAT

Having estimated the CCAT values for all cells, we select the top 5% of cells with highest CCAT values. To robustly identify the root-state, we consider cells in the root state as defining the largest cluster among these 5% of cells, when clustered along *all* the diffusion map components. We estimate diffusion map components using the destiny R-package (Angerer et al., 2016; Haghverdi et al., 2016) and inferred clusters of cells using the walktrap algorithm (Pons and Latapy, 2005) as implemented in the igraph R package (version 1.2.5). This algorithm partitions the diffusion map graph into densely connected modules exploiting the fact that short random walks tend to stay within a module. We set the length of the random walks to be approximately 25% of all cells. For instance, in the application to the liver developmental study (*n* = 447 cells) (Yang et al., 2017), there were 22 high CCAT cells, and so we ran walktrap with a random-walk length of 5. Finally, the root-state is identified as the largest inferred cluster with the root-cell defined as the median cell within this cluster. This root-cell is then used to infer lineage-trajectories using destiny. Details can be found in Supplementary Note.

## 3 Results

### 3.1 CCAT exhibits high discriminative accuracy

**Fig. 1.**
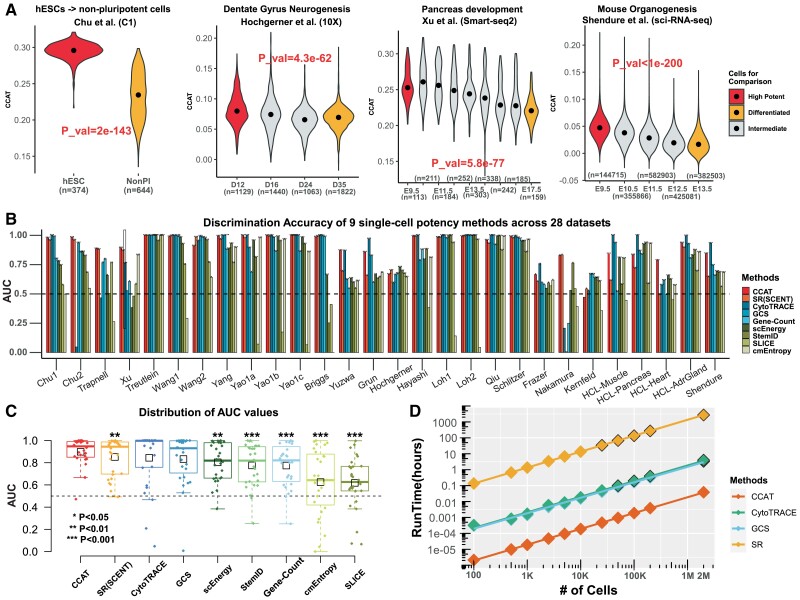
Evaluation and benchmarking of CCAT. (**A**) Distribution of CCAT single-cell potency measure as a function of cell-type or developmental/differentiation timepoint for four independent scRNA-Seq studies. Groups labeled in red and orange denote the cell-types that ought to differ in terms of differentiation potency, with groups labeled in gray denoting intermediate stages. *P*-value is from a one-tailed Wilcoxon rank sum test comparing the higher potency group (red) to the lower potency one (orange). Number of cells in each group is given below violin plots. (**B**) The discriminative accuracy (AUC) derived from the Wilcoxon rank sum test, displayed for 9 different single-cell potency methods across 28 independent scRNA-Seq studies. (**C**) Distribution of the AUC values in (B) for each of the nine single-cell potency estimation methods. Each boxplot contains 28 datapoints represent the 28 studies. Methods have been ranked according to the mean AUC over the 28 studies, indicated by the small black boxes. *P*-values derive from a paired one-tailed Wilcoxon rank sum test comparing the AUC of CCAT to each of the other methods, i.e. with the alternative hypothesis that the AUC for CCAT is higher. We only mark significant *P*-values as shown. *P*-values for the other alternative, i.e. that the AUC of each other method is higher than that of CCAT were all non-significant (*P *>* *0.05). (**D**) Run times (*y*-axis) against the number of cells in a scRNA-Seq study for four different methods, as indicated. Run times were obtained on an Intel Xeon E3-1575M v5/3 GHz and using only 1-core. If run in parallel, say 100 cores, run times can be approximated by dividing values in panel by 100. Data points with black borders were estimated from a linear regression fit

In order to evaluate CCAT, we collated 28 scRNA-Seq datasets, each one profiling cells from distinct differentiation potency states, thus allowing for an objective assessment (Supplementary Table S1). In each dataset, we compared the CCAT values of high to low potency cells, computing the discriminative accuracy (AUC) of CCAT. Of note, in timecourse studies, we only compared cells at the start and endpoints, as these groups of cells ought to differ substantially in terms of differentiation potency, thus allowing AUC to be used as an objective evaluation metric. In total, the 28 comparative studies profiled over 2 million cells, used a wide range of different scRNA-Seq technologies, and encompassed two species (human and mouse). Across all studies, CCAT achieved reasonably high AUC values, with cells in intermediate potency groups also displaying intermediate CCAT values (Fig. 1A and B).

We benchmarked CCAT against eight other single-cell potency methods (Section 2), including SR/SCENT (Teschendorff and Enver, 2017) and CytoTRACE (Gulati *et al.*, 2020), two of the best performing methods in a recent comparative study (Gulati *et al.*, 2020). Overall, CCAT achieved comparable performance to *SR* and CytoTRACE (Fig. 1B and C). In fact, although CytoTRACE was the best performer in terms of the median AUC over all 28 studies, CCAT ranked top in terms of the average AUC, a metric which better assesses the robustness of the method (Fig. 1C). Indeed, we observed that in some studies CytoTRACE broke down, performing less well than some of the other potency measures it is derived from (e.g. number of detected genes or gene-count, Fig. 1B and C). CCAT exhibited statistically significantly higher median AUC compared to all other methods, except CytoTRACE and GCS (gene-count signature) (Fig. 1C), clearly demonstrating that CCAT achieves a discriminative accuracy comparable to that of current state-of-the-art methods. Although in some studies CCAT estimates were highly correlated with GCS, in others, correlations were only moderate, suggesting that these potency measures are not redundant (Supplementary Fig. S1).

### 3.2 CCAT is 100-fold faster than competing methods

Fast estimation of differentiation potency is highly desirable for two reasons. First, estimation of single-cell potency is particularly necessary in scRNA-Seq studies that aim to identify stem-or-multipotent like cells, specially those that underpin homeostasis in adult tissues. Because these stem-or-multipotent progenitor cells are relatively infrequent, their identification requires profiling of large numbers of cells, placing a great computational burden on the computation. Second, normalization of scRNA-Seq data is tricky and computations often need to be redone with different normalization schemes, which means that lengthy computations are undesirable. Since CCAT estimates differentiation potency through a simple Pearson correlation between a cell’s transcriptome and a PPI’s connectome, it is computationally fast. To demonstrate this, we used CCAT to estimate differentiation potency in over two million cells from a mouse organogenesis study (Cao et al., 2019), a computational task that we could accomplish in a few minutes using only 1 Intel Xeon core (Fig. 1D). With 100 cores, runtimes would be a few seconds. In contrast, we estimated that with one core this task would take on the order of hours had we used CytoTRACE or GCS, and a month if using *SR* (Fig. 1D). Overall, CCAT exhibits an approximate 100-fold improvement in computational efficiency as compared to CytoTRACE and GCS.

### 3.3 CCAT identifies a multipotent root-state in scRNA-Seq data

**Fig. 2.**
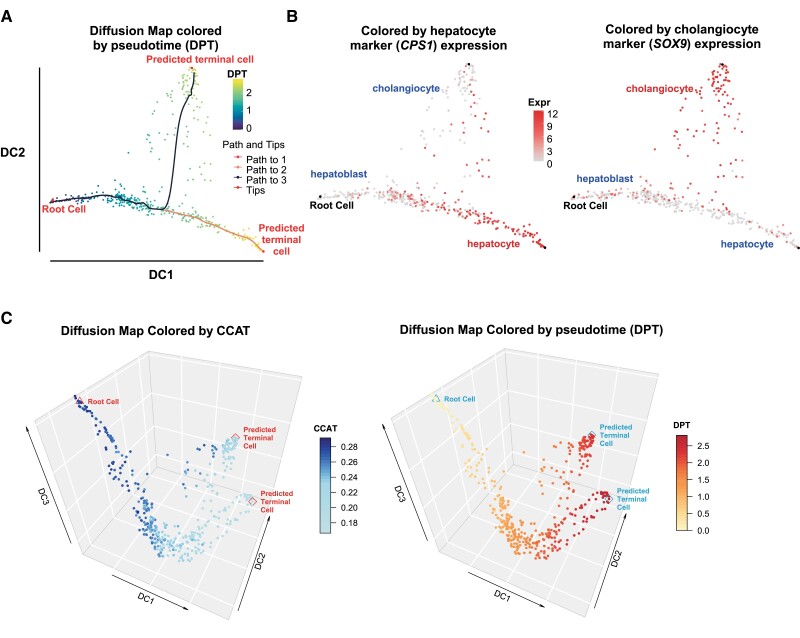
Using CCAT to infer root-state and lineage trajectories in liver development. (**A**) Two-dimensional diffusion map plot displaying the root-cell as identified with CCAT, and the lineage trajectories into hepatocytes and cholangiocytes, with cells colored according to diffusion pseudotime (DPT). (**B**) As (A), but now with cells colored according to *CPS* and *SOX9* expression, which are markers for hepatocytes and cholangiocytes, respectively. (**C**) Three dimensional diffusion map plot, displaying the positioning of the cells along the top 3 DCs, with cells colored by the estimated CCAT values (left panel) and diffusion pseudotime (right panel)

As with *SR* and CytoTRACE, CCAT can be used to unambiguously identify stem-or multipotent root-states, which are necessary for inferring lineage-trajectories. To illustrate this, we estimated potency with CCAT in hundreds of single-cells from a developmental timecourse of hepatoblasts into hepatocytes and cholangiocytes (Yang *et al.*, 2017) and developed a simple algorithm to robustly identify a root-state (and root-cell) among cells attaining high CCAT values (Section 2 and Supplementary Fig. S2). Having identified the root-cell, we next infered lineage trajectories and pseudotime using Diffusion Maps (Angerer *et al.*, 2016; Haghverdi *et al.*, 2016), which revealed a natural bifurcation into hepatocyte and cholangiocyte lineages (Fig. 2A and B). Displaying the diffusion map along the first 3 diffusion components (DCs) further revealed how the third DC associates strongly with potency and CCAT (Fig. 2C).

### 3.4 Robustness of CCAT to PPI network

**Fig. 3.**
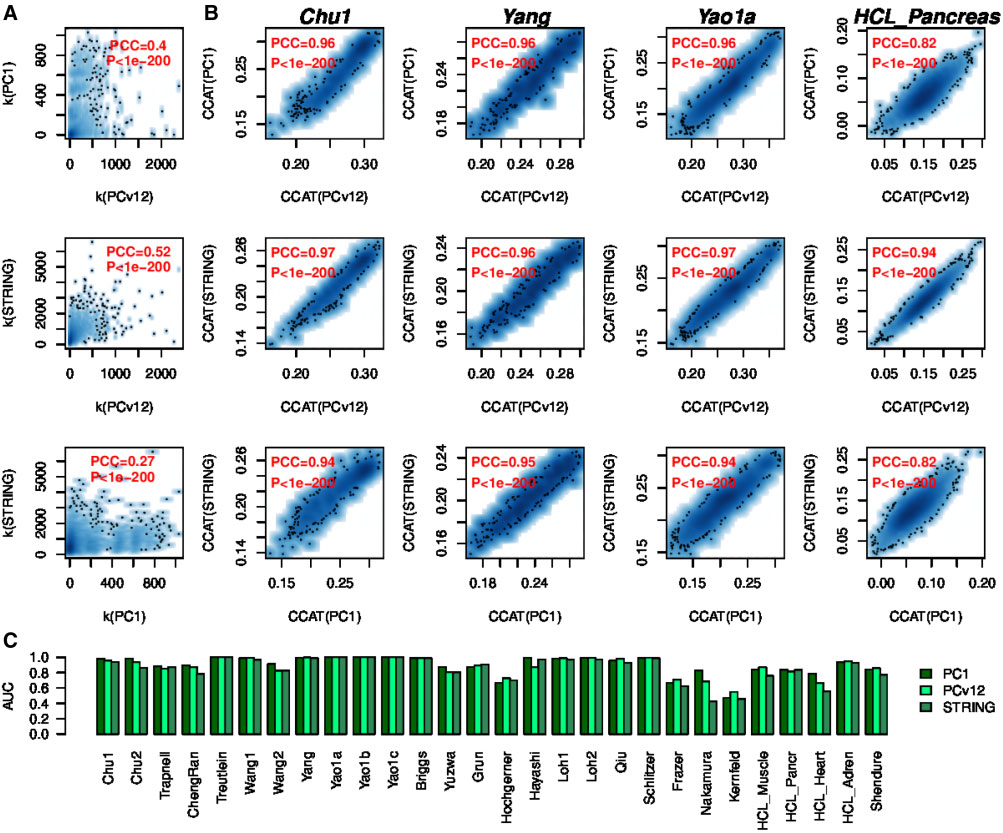
Robustness of CCAT to choice of PPI network. (**A**) Scatterplots of the degree of each protein between each pair of PPI networks (PC1 = Pathway Commons V1, PCv12 = Pathway Commons v12, STRING=STRING database v11). The Pearson Correlation Coefficient (PCC) and associated *P*-value are shown in red. (**B**) Scatterplots of the CCAT estimates derived from each of the three PPI networks and in each of four independent scRNA-Seq studies (Chu1, Yang, Yao1a and HCL-Pancreas). The Pearson Correlation Coefficient (PCC) and associated *P*-value are shown in red. (**C**) Barplots displaying the accuracy (AUC) of CCAT to discriminate high and low potency cells as a function of PPI network used and scRNA-Seq dataset

**Fig. 4.**
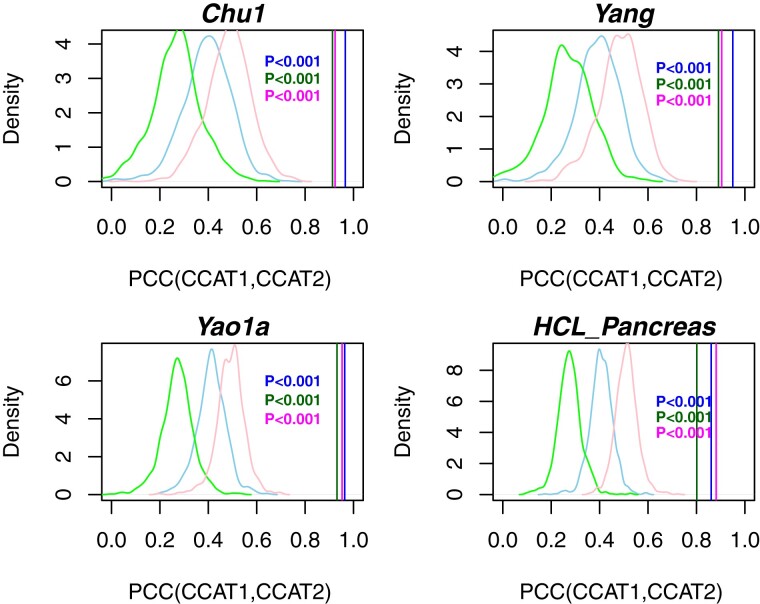
Statistical significance of CCAT correlations. For each of four scRNA-Seq studies (Chu1, Yang, Yao1a and HCL-Pancreas), we compare the Pearson correlation of CCAT values between each pair of PPI network, denoted PCC(CCAT1, CCAT2) (3 vertical lines), to the null distribution (density curves) obtained by permuting the degree distribution of each network (1000 Monte-Carlo runs). Colors label the different pairs of PPI networks: blue (PC1-PCv12), green (PC1-STRING), magenta (PCv12-STRING). Empirical *P*-value is given

It is important to establish the robustness of the CCAT measure to the choice of PPI network. To this end we compared the CCAT values derived from three different PPI networks, encompassing two different versions of the Pathway Commons PPI network (v1 and v12) (Cerami *et al.*, 2011; Rodchenkov *et al.*, 2020) and the latest version of the STRING PPI database (Szklarczyk *et al.*, 2019). Importantly, even though the degree distributions (connectomes) of these networks exhibit moderate correlations (Fig. 3A), CCAT values were highly correlated (Fig. 3B), resulting in excellent discrimination accuracy of high and low potency cells, independently of PPI network and dataset (Fig. 3C). To demonstrate that the increased correlation of CCAT values compared to those of the connectome is statistically significant, we randomized the degree distribution of each PPI network pair, using the same permutation over the common nodes of each network. This permutation scheme guarantees that the correlation of the connectomes is invariant, but destroys any putative association between connectome and transcriptome. This analysis showed that the high correlation of CCAT values is highly significant and not expected by random chance (1000 Monte-Carlo runs *P *<* *0.001, Fig. 4). Thus, the robust association of CCAT with differentiation potency is due to the subtle positive correlation between transcriptome and connectome, which, as shown, is itself robust to the choice of PPI network.

### 3.5 Robustness of CCAT to dropouts

Dropouts are common in scRNA-Seq datasets (Stegle et al., 2015), and several of the datasets analyzed here [e.g. the DropSeq Human Cell Landscape (HCL) datasets] exhibited relatively high (i.e. over 95%) dropout rates (Supplementary Fig. S3). Despite this, measures like CCAT and CytoTRACE exhibited reasonably high discrimination accuracies in these datasets (Fig. 1B). To better assess the effect of an increased dropout rate, we selected 13 studies where CCAT, CytoTRACE and GCS performed well and where the dropout rate in the original data was less than 90%, in order to then be able to evaluate the change in these potency estimates as the dropout rate is increased (Section 2). Across all 13 studies, when the dropout rate in each was increased by 5%, the CCAT measure was the most robust, exhibiting Pearson correlations of over 0.8 across all studies (Supplementary Fig. S4). CCAT was significantly more robust than CytoTRACE and GCS (paired Wilcox test *P* = 0.0006 and *P* = 0.02). Similar results were observed when the dropout rate was increased by 10% (Supplementary Fig. S4). Importantly, discrimination accuracies were robust and remained high for all three methods, although GCS did exhibit some significant variability in two studies (Supplementary Fig. S5).

### 3.6 Stability of CCAT to imputation of dropouts

It is also important to establish robustness of the CCAT measure to imputation of technical dropouts. To this end, we applied the MAGIC imputation algorithm (van Dijk et al., 2018) to four randomly chosen scRNA-Seq datasets, recomputing CCAT and subsequently comparing to the CCAT values without prior imputation. Overall, we observed reasonably strong correlations before and after imputation (Supplementary Fig. S6), with CCAT AUC values also exhibiting strong robustness (Supplementary Fig. S7).

## 4 Discussion

As assessed over 28 RNA-Seq studies, encompassing over 2 million cells, CCAT provides reasonably accurate and robust differentiation potency estimates at single-cell resolution. We did not observe that state-of-the-art methods like CytoTRACE or SR/SCENT outperformed CCAT in terms of accuracy and robustness. Importantly, CCAT offers significant improvements in computational efficiency, yielding a 100-fold speed-up over CytoTRACE or GCS.

We have also demonstrated the robustness of the CCAT measure to the choice of PPI network, to dropouts and to imputation of dropouts. The robustness to the PPI network is particularly noteworthy. Indeed, whilst the correlation of node-degrees between different PPI networks was generally only modest (PCC ∼ 0.5), correlations between CCAT values were generally much higher (PCC > 0.8), thus rendering the ranking of cells according to the CCAT potency measure very robust to the choice of PPI network. As shown by our Monte-Carlo randomization analysis, this robustness stems from the fact that differentiation potency is encoded by a subtle positive correlation between the degree of a gene in a PPI network and its expression level, with highly potent cells exhibiting higher expression of network hubs. It is likely that higher expression of network hubs reflects a more promiscuous interaction pattern of the encoded proteins, facilitated by a more open chromatin structure, in line with the observation that potency and chromatin loosening are correlated (Gulati *et al.*, 2020). Of note, CCAT exhibited a higher robustness to an increase in the dropout rate compared to CytoTRACE and GCS, which is probably driven by the fact that potency is encoded by overexpression of network hubs, where the high expression of such hubs renders CCAT intrinsically more robust to dropouts.

## 5 Conclusion

We have presented an ultra-fast, accurate and robust method for estimating differentiation potency at single-cell resolution from scRNA-Seq data, and which is freely available our SCENT R-package. CCAT should be particularly valuable for scRNA-Seq studies profiling hundreds of thousands to a million cells in complex tissues, to help identify stem-and-multipotent progenitor like cells.

## Funding

A.E.T. was supported by the National Natural Science Foundation of China [31571359, 31771464, 31970632].

*Conflict of Interest*: none declared.

## Supplementary Material

btaa987_Supplementary_Data
