# Supplementary material for: Ultra-fast scalable estimation of single-cell differentiation potency from scRNA-Seq data
Source: Bioinformatics. 2020 Nov 27;37(11):1528–34. doi: 10.1093/bioinformatics/btaa987 (PMC8275983; doi:10.1093/bioinformatics/btaa987)
Supplement: btaa987_Supplementary_Data [file btaa987_supplementary_data.zip › Supplementary Information.pdf]

Supplementary Information for "Ultra-fast scalable estimation of single-cell differentiation potency from scRNA-Seq data"

This document contains Supplementary Methods and Supplementary Figures S1-S7

SUPPLEMENTARY METHODS:

Processing and normalization of scRNA-Seq datasets:
All the scRNA-Seq datasets used in this work are described in Supplementary Table S1, which is provided as a separate excel file. Briefly, the raw counts of each scRNA-seq data matrix were downloaded from the repositories as outlined in Supplementary Table 1. ERCCs spike-ins and genes mapping to mitochondrial genes were removed. Data was then rescaled to transcripts per million (TPM) or counts per million (CPM), and subsequently log2-normalized with a pseudocount of +1, or +1.1 for subsequent SR calculation. The AnnotationDbi v1.48.0 , org.Hs.eg.db v3.10.0 and org.Mm.eg.db v3.10.0 R packages were used to map provided gene IDs to Entrez gene IDs. R- package annotationTools v1.60.0, with "homologene.data" downloaded from https://ftp.ncbi.nih.gov/pub/HomoloGene/current/, with reference HomoloGene v68 (NCBI), was used to get homolog genes when needed, for instance in mouse studies where integration with a human PPI network (CCAT & SR) was necessary.

Software used and implementation:
The following software were applied using default settings. *R packages:* LandSCENT v0.99.3[1][2], SLICE va12242016 [3], StemID v0.2.0 (entropy function only)[4]. *R scripts:* cm-entropy-score [8], CytoTRACE [7]. *Matlab package:* scEpath [6]. Further details:

- **LandSCENT:** The Gene IDs of scRNA-seq data matrices were converted to Human EntrezID before LandSCENT implementation. Three different human protein-protein interaction networks, as specified in the main Methods section, were used to calculate Signaling Entropy Rate (SR) and CCAT.
- **SLICE:** scRNA-seq datasets for human were analyzed with the human gene-gene Kappa similarity matrix, while datasets for mouse were analyzed with the mouse similarity matrix, as provided by the authors.
- **StemID:** The RaceID3_StemID2_package was downloaded from https://github.com/dgrun/RaceID3_StemID2_package. The script named "RaceID3_StemID2_class.R" was used to calculated StemID scores.
- **cmEntropy:** The R script named "entropy_functions.R" and supporting data file named "clean_nodatasets_031020.Rdata" were downloaded from https://github.com/skannan4/cm-entropy-score.
- **CytoTRACE:** The R script named "CytoTRACE.R" was downloaded from the website https://cytotrace.stanford.edu, provided by the authors. We collected both CytoTRACE and GCS (Gene Count Signature) values.
- **scEpath:** The Matlab package are downloaded from https://github.com/sqjin/scEpath. For methods comparison convenience, we translated the core entropy functions into R code and only use the R code to implement this method.

Run-time analysis

We compared CCAT to SR, GCS and CytoTRACE in terms of their computational efficiency. Without loss of generality we took the HCL_AdrenalGland scRNA-Seq dataset, composed of over 20,000 genes and over 16,000 cells. We then generated scRNA-Seq datasets with variable cell numbers *n* (*n*=100, 500, 1000, 2500, 5000, 10000, 25000, 50000, 100k, 200k and 2 million), by subsampling in the case *n < 16,000* or by sampling with replacement when *n>16,000.* In order to avoid confounding by server-load, we ran all methods using only 1-core (Intel Xeon E3-1575M v5/3GHz) on a Dell Mobile Workstation with 64 GB RAM. Because computing SR can be computationally demanding, we only did this for cell numbers up to 10,000. For CCAT we obtained direct estimates for all considered cell-numbers. In the case of the larger data matrix with 2 million cells, we avoided the use of memory-efficient dgC-class matrices (which can slow computations), by splitting the data-matrix up into 10 chunks of 200,000 cells each. We note that since SR and CCAT compute the potency of each cell without using information from other cells, that these methods are truly linear in the number of cells. For CytoTRACE and GCS we obtained run-times for cell-numbers up to 25,000. Because these two methods are defined by running on all available genes, and not just those that overlap with a PPI network, we found that they broke down when the cell-number was 50k or higher, due to memory constraints. We did not want to use dgC-class matrices, because this was not make it comparable to runtimes obtained at smaller cell-numbers. Hence, for CytoTRACE and GCS we estimated run-times for *n=50k, 100k, 200k* and *2m,* by fitting a regression line to the run-times obtained for the smaller cell-numbers. We observed that for CytoTRACE and GCS, run-times also scaled linearly with cell-number, justifying the estimations. All directly obtained run-times were obtained by averaging over 10 Monte-Carlo runs, which was sufficient to yield accurate/reproducible run-times. We note that in the case of GCS we modified the R-script CytoTRACE.R to remove all the computations subsequent to the calculation of the GCS-score. Thus, run-times for GCS are marginally lower than for CytoTRACE.

Robustness to dropouts:

While many of the scRNA-Seq studies exhibited relatively high dropout rates (>95%), well-performing methods on these datasets already indicate a level of robustness, we also evaluated the robustness of methods to increases in dropout rates. This was done for the three best performing methods (CCAT, GCS and CytoTRACE) and for scRNA-Seq studies where (i) all 3 methods performed well (AUC>0.8), and (ii) where the dropout rate of the study was <90%. We then increased the dropout rate by 5 and 10% and recomputed the CCAT, GCS and CytoTRACE estimates. Robustness was then assessed by Pearson Correlation Coefficients and recomputing the AUC between high and low potency cells.

Robustness of CCAT to imputation of dropouts:

We tested robustness of CCAT values to imputation of dropouts. To impute dropouts we used MAGIC [5] (Rmagic v2.0.3 R package). Rmagic was applied to the normalized log-transformed data (using pseudocount of 1). As required by MAGIC, PCA dimensionality reduction was done so as to retain ~70% of the variation in the data.

SUPPLEMENTARY FIGURES:

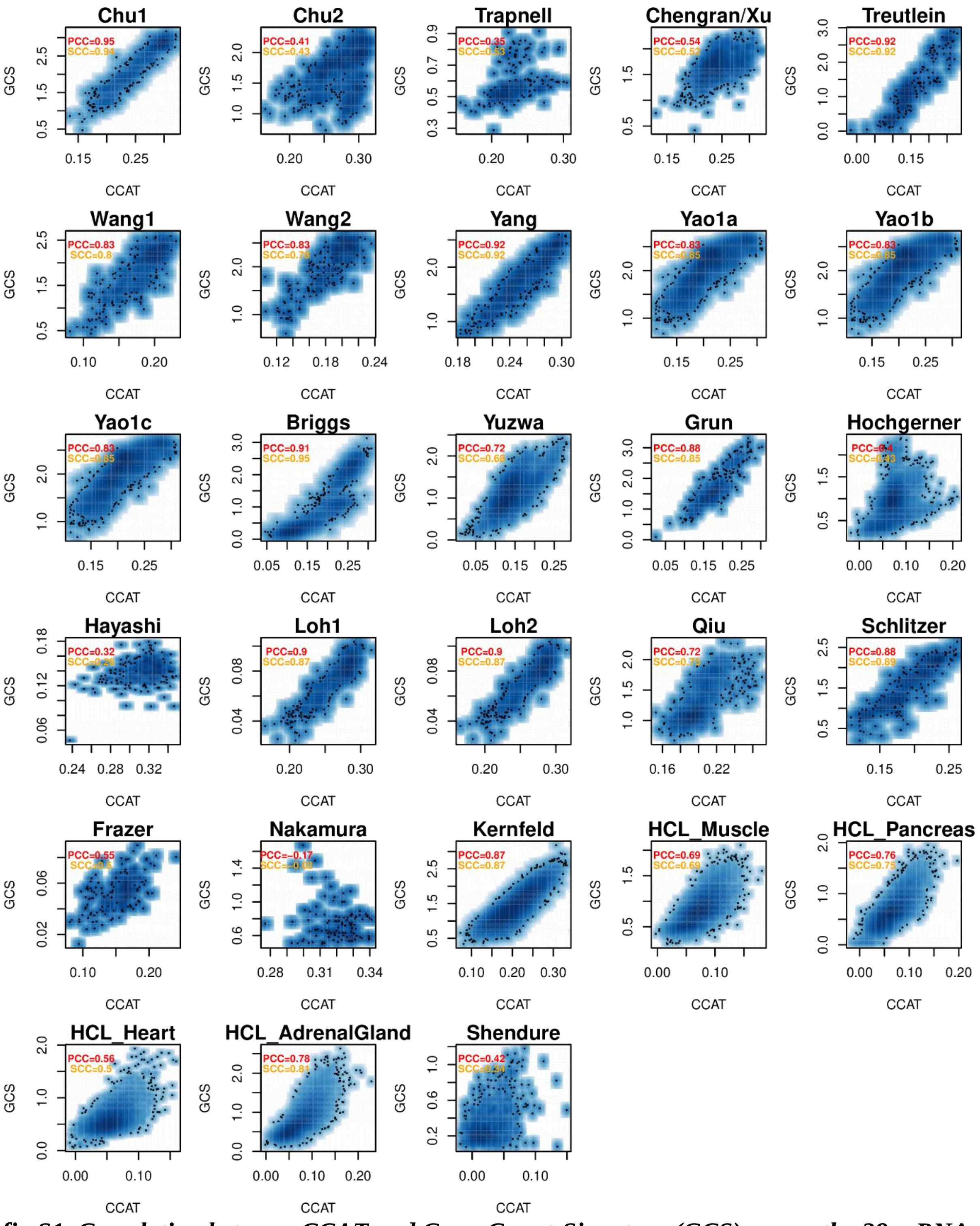

fig.S1. Correlation between CCAT and Gene-Count-Signature (GCS) across the 28 scRNA-Seq studies: *Smoothed scatterplots of GCS and CCAT scores for each of the 28 scRNA-Seq datasets considered here. Pearson correlation and Spearman correlation coefficients (PCC & SCC) are given.*

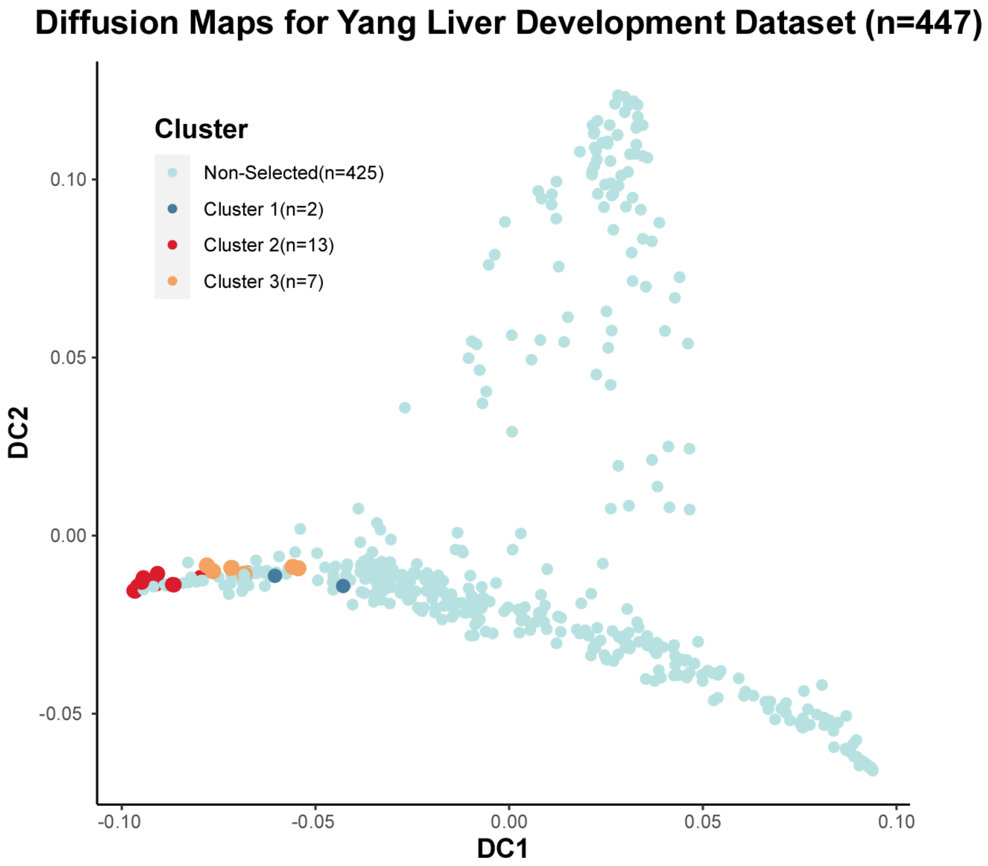

fig.S2. Selection of root-state & root-cell from CCAT in liver development study: *Diffusion map plot displaying the top 2 diffusion components (DCs) as derived using destiny diffusion maps to a liver developmental scRNA-Seq time course. The 5% of cells with highest CCAT values (22 cells in total) are colored according to the result of the walk-trap clustering algorithm, which clustered these 22 cells into 3 clusters, as shown. The root-state is identified as the largest cluster, i.e. cluster-2 (red), and the root-cell is defined as the median cell in cluster-2.*

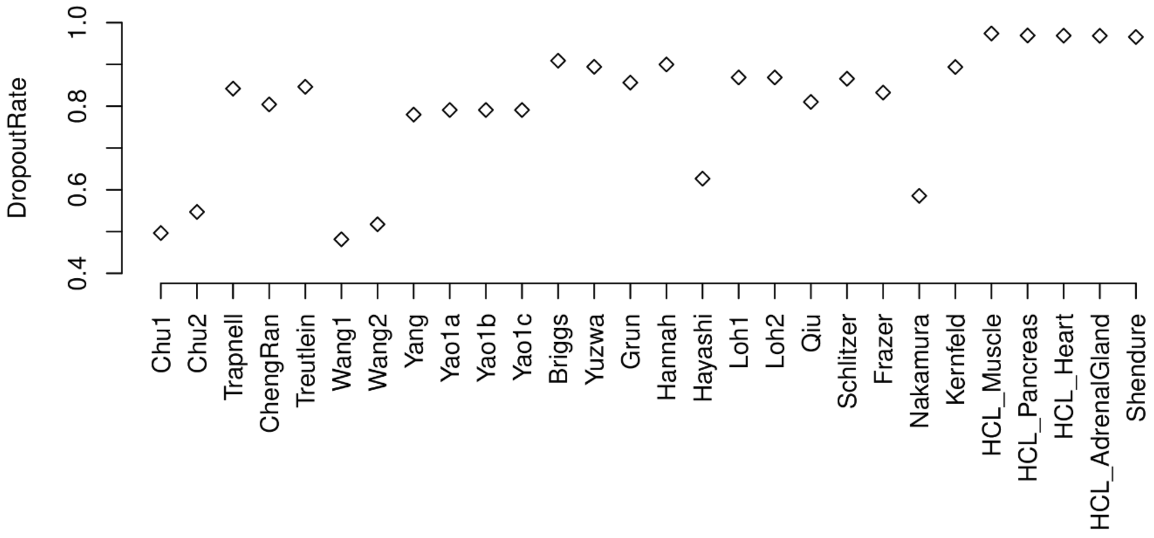

fig.S3. Dropout rates across the 28 scRNA-Seq studies analysed here: *The dropout rate, defined as the fraction of zeroes, is displayed against the scRNA-Study, as shown.*

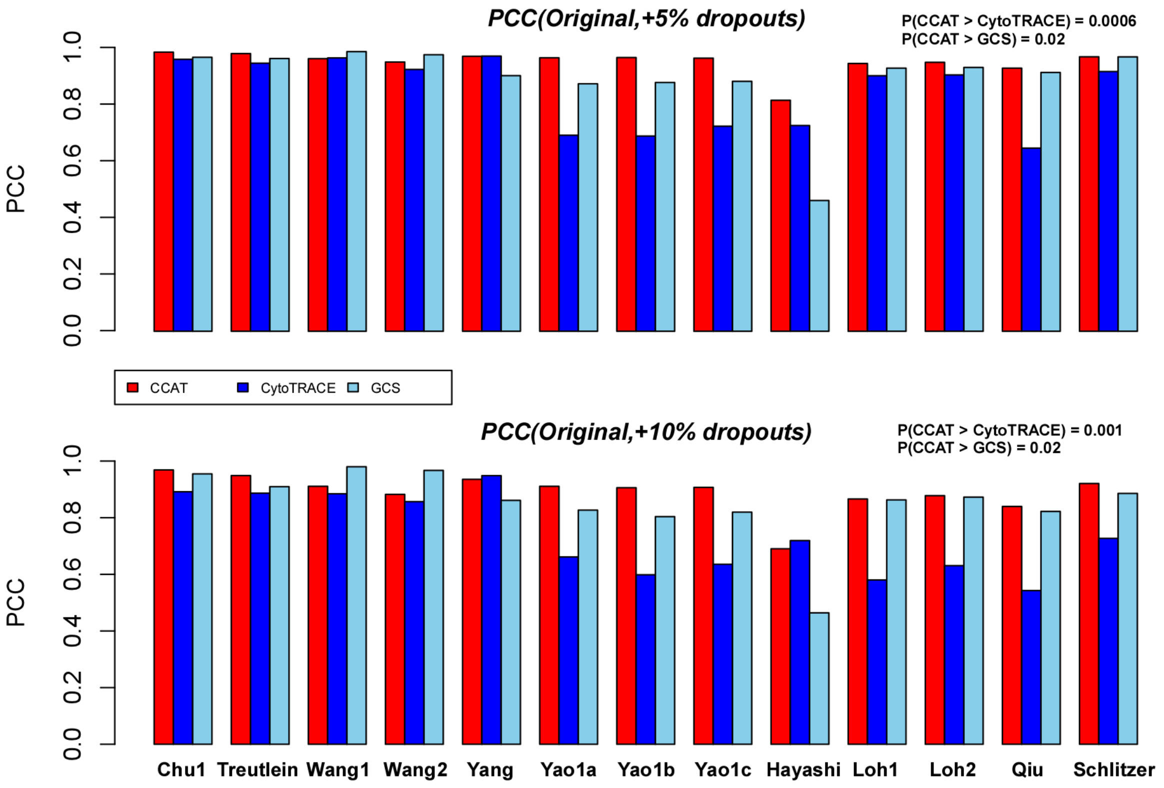

fig.S4. Robustness of CCAT, CytoTRACE and GCS to an increased dropout rate: *For each of 13 scRNA-Seq datasets, where the original dropout rate was less than 90%, we display the Pearson Correlation Coefficient (PCC) between the potency estimate derived from the original data, and the potency estimate obtained after introducing an additional 5% (top panel) and 10% (bottom panel) dropouts. PCCs are shown for 3 different potency measures: CCAT, CytoTRACE and GCS. The P-value from a one-tailed paired Wilcoxon rank sum test, comparing CCAT to CytoTRACE and GCS is given with the alternative hypothesis being that CCAT exhibits higher PCC values.*

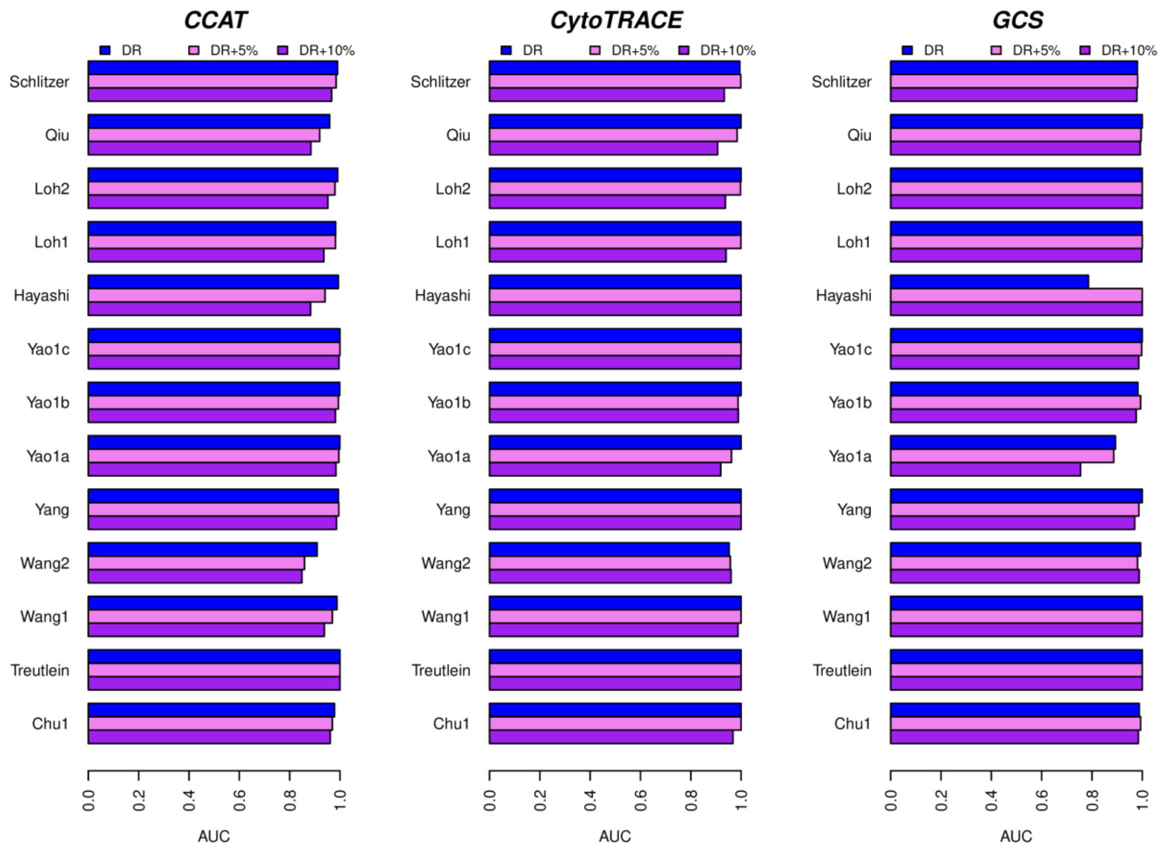

fig.S5. Robustness of the discrimination accuracy (AUC) of CCAT, CytoTRACE and GCS to an increased dropout rate: *For each of 13 scRNA-Seq datasets (y-axis), where the original dropout rate (DR) was less than 90% and where all 3 methods performed well (AUC>0.8), we display the AUC (x-axis) between high and low potency cells for the original data (DR) and for increases of 5 and 10% in the original dropout rate.*

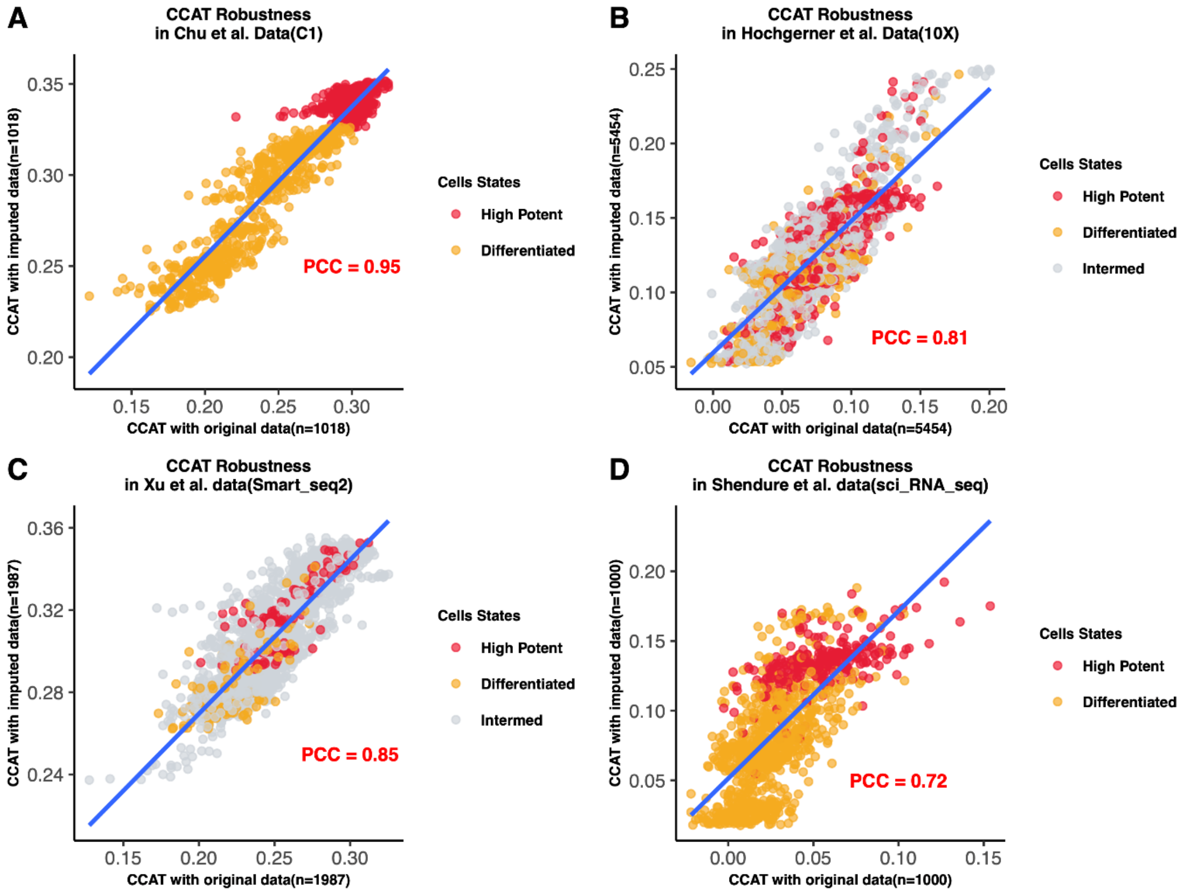

fig.S6. Robustness of CCAT measure to imputation of dropouts: A) *Scatterplot of CCAT values, before (x-axis) and after imputation (y-axis) in the Chu et al dataset. Blue line is the least squares regression line. The Pearson correlation coefficient is given in red text. The Chu et al. dataset contains hESCs (red data points) and non-pluripotent multipotent progenitor cell-types (orange data points).* ***B-D)*** *As A), but for three other datasets: Hochgerner et al. describes postnatal dentate gyrus neurogenesis, Xu et al. dataset is a time course mouse pancreatic differentiation process, which is from progenitors to differentiated beta and alpha cells, and the Shendure et al. dataset describes mouse organogenesis during 5 embryo stages, here we only selected 1000 cells for comparison.*

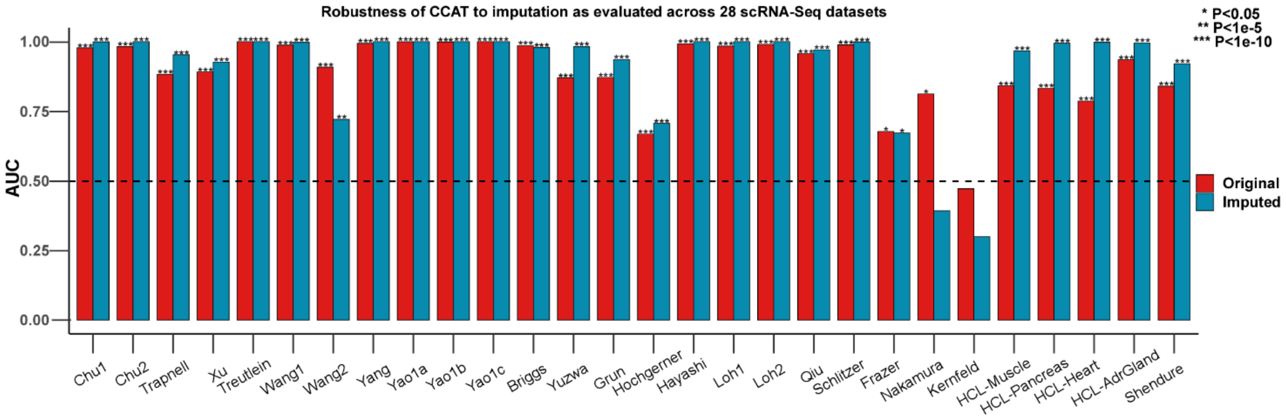

fig.S7. Robustness of CCAT's discriminative accuracy to imputation: *Barplot of AUC values, before and after imputation, across the 28 comparisons. Significance of AUC values is indicated, with P-values derived from a one-tailed Wilcoxon rank sum test.*

Supplementary References:

1. Teschendorff, A., Enver, T. Single-cell entropy for accurate estimation of differentiation potency from a cell's transcriptome. Nat Commun 8, 15599 (2017). https://doi.org/10.1038/ncomms15599
2. Chen, W., Morabito, S.J., Kessenbrock, K. et al. Single-cell landscape in mammary epithelium reveals bipotent-like cells associated with breast cancer risk and outcome. Commun Biol 2, 306 (2019). https://doi.org/10.1038/s42003-019-0554-8
3. Guo, E. L. Bao, M. Wagner, J. A. Whitsett, Y. Xu, SLICE: Determining cell differentiation and lineage based on single cell entropy. Nucleic Acids Res. 45, e54 (2017). 10.1093/nar/gkw1278
4. D. Grun, M. J. Muraro, J.-C. Boisset, K. Wiebrands, A. Lyubimova, G. Dharmadhikari, M. van den Born, J. van Es, E. Jansen, H. Clevers, E. J. P. de Koning, A. van Oudenaarden, De novo prediction of stem cell identity using single-cell transcriptome data. Cell Stem Cell 19, 266–277 (2016). doi:10.1016/j.stem.2016.05.010
5. van Dijk D, Sharma R, Nainys J, et al. Recovering Gene Interactions from Single-Cell Data Using Data Diffusion. Cell. 2018;174(3):716-729.e27. doi:10.1016/j.cell.2018.05.061
6. Jin S, MacLean AL, Peng T, Nie Q. scEpath: energy landscape-based inference of transition probabilities and cellular trajectories from single-cell transcriptomic data. Bioinformatics. 2018;34(12):2077-2086. doi:10.1093/bioinformatics/bty058
7. Gulati GS, Sikandar SS, Wesche DJ, et al. Single-cell transcriptional diversity is a hallmark of developmental potential. Science. 2020;367(6476):405-411. doi:10.1126/science.aax0249
8. Kannan S, Farid M, Lin BL, Miyamoto M, Kwon C: Transcriptomic entropy quantifies cardiomyocyte maturation at single cell level. . bioRxiv 2020, 2020.04.02.022632.
