# Supplementary material for: Ultra-fast scalable estimation of single-cell differentiation potency from scRNA-Seq data
Source: Bioinformatics. 2020 Nov 27;37(11):1528–34. doi: 10.1093/bioinformatics/btaa987 (PMC8275983; doi:10.1093/bioinformatics/btaa987)
Supplement: btaa987_Supplementary_Data [file btaa987_supplementary_data.zip › TutorialSCENT.pdf]

A brief tutorial for estimating differentiation potency of single cells using SCENT and CCAT

Andrew E. Teschendorff [1,2]

[1]CAS Key Lab of Computational Biology, PICB, SINH
[2]UCL Cancer Institute, University College London

2020-10-26

Package

SCENT 1.0.2

Contents

1 Introduction
2 The functional gene network
3 Single cell RNA-seq data
4 Differentiation potency estimation using Signaling Entropy Rate (SR)
5 Differentiation potency estimation using CCAT
6 Infer the potency states in a cell population
7 Integration with diffusion maps to infer differentiation trajectories
8 Session information
References

1 Introduction

The main purpose of the `SCENT` package is to provide a means of estimating the differentiation potency of single cells without the need to assume prior biological knowledge such as marker expression or timepoint. This may be particularly important in scenarios where a high dropout rate may preclude the use of a marker gene, in snapshot scRNA-Seq datasets of complex tissues where differentiation hierarchies are not well-established, or in cancer tissue where one may want to identify putative cancer stem-cell phenotypes.

This document gives a detailed tutorial on how to use `scent` to estimate differentiation potency of single-cells from scRNA-Seq data. The following inputs are needed:

- Normalized single cell RNA-seq data
- A functional gene network

How to prepare these inputs for using in `SCENT` will be described in detail in the following sections.

2 The functional gene network

In `SCENT`, the estimation of differentiation potency of single-cells can be done in two different ways: (i) using the SCENT algorithm which approximates differentiation potency in terms of the entropy of a diffusion process on a gene network, also called signaling entropy rate (Andrew E Teschendorff and Enver 2017), or (ii) via a fast proxy of SCENT called CCAT (Correlation of Connectome and Transcriptome). The latter approach uses the same network as in SCENT, but only uses the connectivity or degree of the genes in the network. The gene-network we recommend to use is a protein-protein interaction (PPI) network, because more potent cells tend to overexpress protein network hubs (Andrew E Teschendorff and Enver 2017) . Indeed, although PPI networks are mere caricatures of the underlying signaling networks, ignoring time, spatial and biological contexts, our approach is to use the measured gene-expression profile of a cell to provide the biological context, in effect weighting the edges in the PPI network according to how highly expressed the proteins in the edge-pair are. For details we refer the reader to our publications given at the end of this vignette(Andrew E Teschendorff and Enver 2017) and (Andrew E Teschendorff, Sollich, and Kuehn 2014). The specific PPI network we use here is derived from Pathway Commons, which is an integrated resource collating together PPIs from several distinct sources. In particular, the network is constructed by integrating the following sources: the Human Protein Reference Database (HPRD), the National Cancer Institute Nature Pathway Interaction Database (NCI-PID), the Interactome (Intact) and the Molecular Interaction Database (MINT).

Within the `SCENT` package we provide two PPI networks, both derived from PathwayCommons, corresponding to two different versions under filenames "net17Jan16.rda" and "net13Jun12.rda"(early version). You can access these with the `data` function. Here we use the early version network:

```
library(SCENT);
data(net13Jun12);
print(dim(net13Jun12.m));
```

```
## [1] 8434 8434
```

We can see that the PPI network contains over 8000 proteins/genes. Importantly, the nodes (genes) in this PPI network are labeled with Entrez gene IDs, and entries take on values "0" and "1", with "0" indicating that there is no interaction or connection between the two genes, and "1" indicating that an interaction has been reported. It is also important to note that the diagonal entries are set to "0".

3 Single cell RNA-seq data

We shall assume that we already have a matrix containing expression count data summarised at the level of genes and that quality control has been performed removing poor quality cells. We shall further assume that the scRNA-Seq data has been normalized for library size using cell-specific scale factors, for instance as implemented in the `scater` R-package. **Importantly, however, the data has not yet been log-transformed.** We shall soon see why.

For ease of illustration, we shall first use a scRNA-Seq dataset from (Chu et al. 2016), generated with the Fluidigm C1 platform. This scRNA-Seq dataset profiled over 1000 cells including pluripotent hESCs and non-pluripotent progenitor cells from the 3 main germ layers (ectoderm, mesoderm and endoderm). Due to package size restriction, we only upload a subset of 479 cells (374 are pluripotent and 105 are multipotent progenitors of endothelial cells). Below we load in the data:

```
data(dataChu)
print(dim(scChu.m));
```

```
## [1] 18935   479
```

```
print(summary(factor(phenoChu.v)));
```

```
##   EC hESC
##  105  374
```

If the user is interested, the full dataset can be downloaded from the GEO website under accession number GSE75748, and the specific file to download is the supplementary file "GSE75748_sc_cell_type_ec.csv.gz". Note that this data matrix has not been normalized so the user would need to do QC and library-size normalization separately using his/her favourite package.

Log-transforming the library-size normalized scRNA-Seq data matrix is important to stabilize the variance of highly expressed genes. Because SCENT uses ratios of expression values it is important to avoid 0 values after log-transformation. For this reason, we use a pseudocount of +1.1 instead of +1. This particular offset ensures that the minimum value after log-transformation is greater than zero. However, for CCAT, 0 values in the data matrix are allowed, so below we also define a separate log-transformed data matrix using the usual pseudocount of +1. This is convenient to retain the sparse nature of the data matrix if it happens to be of the dgC-class (see `Matrix` package).

```
lscChu.m <- log2(scChu.m+1.1);
range(lscChu.m);
```

```
## [1]  0.1375035 16.5819775
```

```
lscChu0.m <- log2(scChu.m+1);
range(lscChu0.m);
```

```
## [1]  0.00000 16.58198
```

We note that the rownames of the data matrix are also annotated to Entrez gene IDs, i.e. the same gene identifier used in the PPI network. If the gene identifiers are different, say if the count-matrix is annotated to gene-symbols, **then these must be converted to unique Entrez gene IDs before proceeding**.

4 Differentiation potency estimation using Signaling Entropy Rate (SR)

The estimation of differentiation potency consists of two major steps:

- Integration of the scRNA-Seq data with the user-defined gene functional network.
- Computation of the Signaling Entropy Rate (denoted SR) which is used to approximate differentiation potency of single cells.

A typical workflow starts with the integration of the scRNA-Seq data with the user-defined gene functional network. This is accomplished with the `DoIntegPPI` function:

```
integ.l <- DoIntegPPI(exp.m = lscChu.m, ppiA.m = net13Jun12.m)
str(integ.l)
```

```
## List of 2
##  $ expMC: num [1:8393, 1:479] 0.138 9.374 4.464 11.055 10.152 ...
##   ..- attr(*, "dimnames")=List of 2
##   .. ..$ : chr [1:8393] "1510" "10436" "7917" "4173" ...
##   .. ..$ : chr [1:479] "H1_Exp1.001" "H1_Exp1.002" "H1_Exp1.003" "H1_Exp1.00
4" ...
##  $ adjMC: num [1:8393, 1:8393] 0 1 1 0 0 0 0 0 0 0 ...
##   ..- attr(*, "dimnames")=List of 2
##   .. ..$ : chr [1:8393] "1510" "10436" "7917" "4173" ...
##   .. ..$ : chr [1:8393] "1510" "10436" "7917" "4173" ...
```

The `DoIntegPPI` function integrates the expression data with the PPI network, and extracts the maximally connected subnetwork, specified by the *adjMC* output argument. In addition, it returns the scRNA-Seq data matrix, defined for the overlapping genes, as specified by the *expMC* output argument.

With the output object *integ.l*, we can now proceed to compute the SR values for any given cell, using the `CompSRana` function. It takes three objects as input:

- The output object *integ.l* from `DoIntegPPI` function
- *local*, a logical parameter to tell the function whether to report back the normalized local, i.e. gene-centric, signaling entropies. This can be useful if one is interested in identifying genes that drive potency. See (Andrew E Teschendorff and Enver 2017).
- *mc.cores*, the number of cores to use, i.e. at most how many child processes will be run simultaneously.

```
sr.o <- CompSRana(integ.l, local = FALSE, mc.cores = 4)
```

The output argument *sr.o* is a list with the following output elements:

- *SR*: the SR value of the cells.
- *inv*: a matrix specifying the invariant measures, or steady-state probabilities, for each cell. That is, each column labels a cell (sample) and is of length equal to the number of nodes in the *adjMC* matrix, with its entries adding to 1.
- *s*: a matrix containing the unnormalized local signaling entropies, and therefore row number equals the number of nodes in the *adjMC* matrix, column number equals the number of cells.
- *ns*: if local=TRUE, a matrix containing normalized local signaling entropies.

One note with the above step: the local gene-based entropies can be used in downstream analyses for ranking genes according to differential entropy, but only if appropriately normalized. For instance, they could be used to identify the main genes driving changes in the global signaling entropy rate of the network. However, if the user only wishes to estimate potency, specifying *local=FALSE* is fine, which will save some RAM on the output object, which is why we make it the default option.

Because the calculation of SR takes a couple of minutes, we use the precomputed values stored in the vector `srChu.v` which is part of `dataChu`. We can now check if SR discriminates hESCs from the multipotent progenitor endothelial cells:

```
boxplot(srChu.v ~ phenoChu.v, main = "SR potency estimates", xlab = "Cell Type"
, ylab = "SR")
```

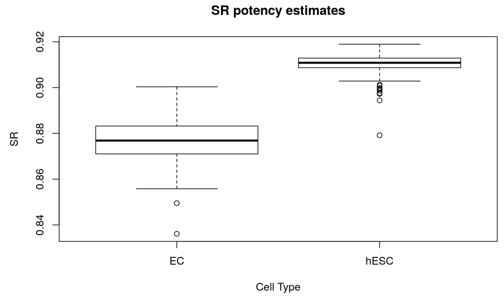

Here *hESC* and *EC* refer to human embryonic stem cells and progenitors of endothelial cells, respectively. Of note, the SR values are normalized to be between 0 and 1, with higher SR values indicative of higher potency.

5 Differentiation potency estimation using CCAT

CCAT is a fast proxy for SR, which we strongly recommend for use on larger scRNA-Seq datasets profiling on the order of ten or hundred thousand cells, or higher (millions of cells). The procedure for estimating differentiation potency using CCAT is similar to SR.

```
ccat.v <- CompCCAT(exp = lscChu0.m, ppiA = net13Jun12.m);
```

We can now check if CCAT discriminates cells according to their potency:

```
boxplot(ccat.v ~ phenoChu.v, main = "SR potency estimates", xlab = "Cell Type",
ylab = "SR")
```

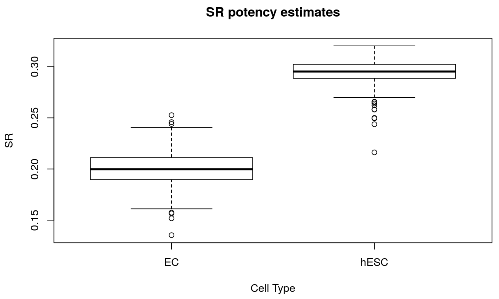

Because CCAT is just a Pearson Correlation Coefficient between the connectome and transcriptome of a cell, it can take on values between -1 and 1, with increasing values indicating higher potency.

6 Infer the potency states in a cell population

Having estimated the cell potency values, it may be of interest to determine if these values cluster into various potency states. This can be accomplished with the `InferPotencyStates` function, which fits a mixture of Gaussians to logit-transformed SR values, or Z-transformed CCAT values. If a categorical phenotype or labeling information of cells is available, the function will return the distribution of inferred potency states in each stratum defined by the phenotype. For instance, we could use the known hESC/EC label:

`r pot.o <- InferPotencyStates(potest.v=srChu.v, pheno.v = phenoChu.v)`

`## [1] "Fit Gaussian Mixture Model to logit-transformed SR values"  ## fitting ...  ##     |`

and the distribution of potency states is:

```
pot.o$distr
```

```
##        ordpotS.v
## pheno.v   1   2
##    EC     0 105
##    hESC 364  10
```

Thus, we have inferred 2 potency states, with the first potency state being occupied by 364 human embryonic stem cells (hESC), while the second lower potency state is enriched for multipotent progenitors of endothelial cells (ECs). Of note, SR predicts a small number of hESCs that have lower potency, representing primed states (Andrew E Teschendorff and Enver 2017).

7 Integration with diffusion maps to infer differentiation trajectories

Potency estimates obtained using SR or CCAT can be integrated with diffusion maps from the R-package `destiny` to assign the root-states when inferring lineage trajectories. We illustrate this in the context of a scRNA-Seq dataset representing a differentiation timecourse of mouse hepatoblasts into hepatocytes and cholangiocytes from (Yang et al. 2017). First, we load in the data:

`r data(dataLiver); dim(scLiver.m);`

`## [1] 16119   447`

The scRNA-Seq has already been normalized for library size, log-transformed, and with genes mapped to 16119 unique human homologs. In total there are 447 cells, from seven different time points (embryonic days 10 to 15, and day 17). To see the distribution of cells among these timepoints:

```
summary(factor(phenoLiver.v));
```

```
##  1  2  3  4  5  6  7
## 54 70 41 65 70 77 70
```

Next, we compute the CCAT values and study its pattern of variation across timepoints

```
ccat.v <- CompCCAT(exp = scLiver.m, ppiA = net13Jun12.m);
boxplot(ccat.v ~ names(phenoLiver.v),xlab="Embryonic stages",ylab="CCAT",col=co
lorLiver.v);
```

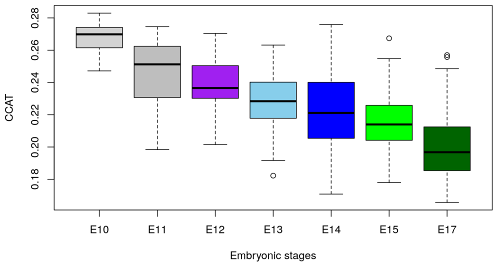

As we can see, CCAT predicts high potency for hepatoblasts at E10, and low potency for cells at the end of the timecourse, as required.

Next, we want to see how we can use CCAT to inform the choice of root-state for inferring lineage trajectories. While in principle we could pick the cell with highest CCAT value as root-state, we strive to provide a method that is more robust. This is achieved by the function `InferDMAPandRoot`.

Briefly, in this method, we use the `destiny` package to construct the diffusion map and Markov Chain transition matrix over all cells. Subsequently, we apply the walktrap clustering algorithm on the inferred graph transition matrix, but only using the cells of highest potency. The proportion of highest potency cells is determined by the `pctop` argument in the function. The walktrap algorithm learns modules within the graph specified by the Markov transition matrix, and we deem the largest cluster to represent a robust root-state. The root-cell is then identified as the cell within the root-state that is closest to the medoid of the cluster. We now run the function:

```
inf.o <- InferDMAPandRoot(pot=ccat.v, exp = scLiver.m, kDMAP=30, pctop=0.05);
```

```
## [1] "The root-state will be inferred from the top 22 cells with highest pote
ncy"
## [1] "The diffusion map will be constructed from 8331 variable genes"
## finding knns......done. Time: 4.07s
## Calculating transition probabilities......done. Time: 0.09s
##
## performing eigen decomposition......done. Time: 0.04s
## [1] "Running walk-trap community detection with max.step number= 5"
## [1] "Walk-trap identified a root-state consisting of 13 cells"
```

In order to check the output, we first plot the distribution of cells along the top 2 diffusion components, coloring cells by timepoint, and indicating the root-cell with a red square box:

```
plot(inf.o$dc[,1],inf.o$dc[,2],col=colorLiver.v[phenoLiver.v],xlab="DC1",ylab="
DC2",axes=FALSE,main="Cells colored by timepoint")
points(inf.o$dc[inf.o$root,1],inf.o$dc[inf.o$root,2],pch=22,col="red",cex=2)
text(inf.o$dc[inf.o$root,1],inf.o$dc[inf.o$root,2],label="Root",font=2,col="red
",pos=1)
par(xpd=TRUE);
legend(x=inf.o$dc[inf.o$root,1],y=0.1,levels(factor(names(phenoLiver.v))),pch=2
1,col=colorLiver.v,cex=.75);
```

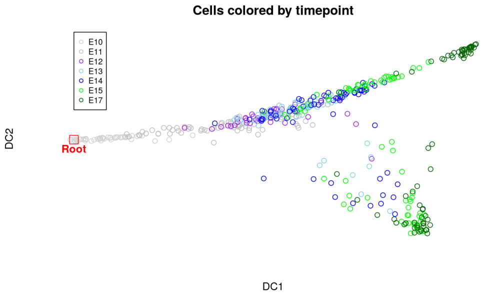

We next display the same plot, but now coloring cells by CCAT value:

```
plot(inf.o$dc[,1],inf.o$dc[,2],col=inf.o$color,xlab="DC1",ylab="DC2",axes=FALSE
,main="Cells colored by CCAT");
points(inf.o$dc[inf.o$root,1],inf.o$dc[inf.o$root,2],pch=22,col="red",cex=2);
text(inf.o$dc[inf.o$root,1],inf.o$dc[inf.o$root,2],label="Root",font=2,col="red
",pos=1)
par(xpd=TRUE);
legend(x=inf.o$dc[inf.o$root,1],y=0.1,c("High","Low"),pch=21,col=c("black","sky
blue"));
```

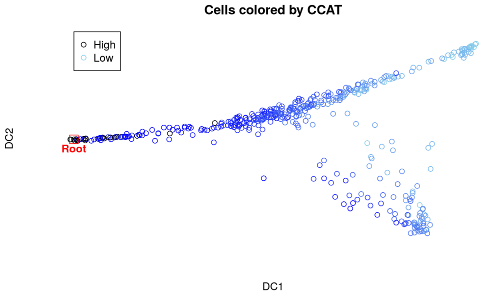

This confirms that the root-cell is a cell of very high potency as estimated using CCAT. Having inferred the root-cell, we can now estimate the diffusion pseudotime (DPT) and plot the inferred lineage trajectories:

```
dptCCAT.o <- destiny::DPT(inf.o$dmap,tips=inf.o$root);
destiny::plot(dptCCAT.o,dcs=1:2,col_by="dpt",paths_to=1:3,pch=23,col_path=marray
::maPalette(low="darkgreen",mid="green",high="yellow",k=3),lwd=3);
```

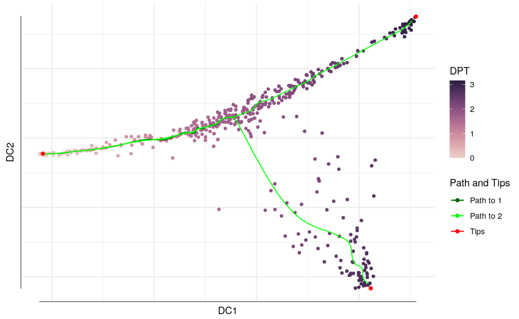

Thus, there are two main trajectories that start at the root-cell, with one trajectory describing differentiation into hepatocytes and another describing differentiation into cholangiocytes, see (A. E. Teschendorff and Wang 2020).

8 Session information

```r
sessionInfo()
```

```
## R version 3.6.3 (2020-02-29)
## Platform: x86_64-pc-linux-gnu (64-bit)
## Running under: Ubuntu 18.04.5 LTS
##
## Matrix products: default
## BLAS:   /usr/lib/x86_64-linux-gnu/blas/libblas.so.3.7.1
## LAPACK: /usr/lib/x86_64-linux-gnu/lapack/liblapack.so.3.7.1
##
## locale:
##  [1] LC_CTYPE=en_GB.UTF-8       LC_NUMERIC=C
##  [3] LC_TIME=en_GB.UTF-8        LC_COLLATE=en_GB.UTF-8
##  [5] LC_MONETARY=en_GB.UTF-8    LC_MESSAGES=en_GB.UTF-8
##  [7] LC_PAPER=en_GB.UTF-8       LC_NAME=C
##  [9] LC_ADDRESS=C               LC_TELEPHONE=C
## [11] LC_MEASUREMENT=en_GB.UTF-8 LC_IDENTIFICATION=C
##
## attached base packages:
## [1] stats     graphics  grDevices utils     datasets  methods   base
##
## other attached packages:
## [1] SCENT_1.0.2      BiocStyle_2.12.0 knitr_1.28       rmarkdown_2.2
##
## loaded via a namespace (and not attached):
##  [1] bitops_1.0-6                matrixStats_0.56.0
##  [3] destiny_2.14.0              xts_0.12-0
##  [5] GenomeInfoDb_1.20.0         docopt_0.6.1
##  [7] tools_3.6.3                 R6_2.4.1
##  [9] BiocGenerics_0.32.0         colorspace_1.4-1
## [11] nnet_7.3-14                 sp_1.4-2
## [13] tidyselect_1.1.0            smoother_1.1
## [15] curl_4.3                    compiler_3.6.3
## [17] Biobase_2.44.0              DelayedArray_0.10.0
## [19] labeling_0.3                slam_0.1-47
## [21] bookdown_0.19               scales_1.1.1
## [23] DEoptimR_1.0-8              lmtest_0.9-37
## [25] robustbase_0.93-6           proxy_0.4-24
## [27] stringr_1.4.0               digest_0.6.25
## [29] foreign_0.8-72              sparsesvd_0.2
## [31] rio_0.5.16                  XVector_0.24.0
## [33] pkgconfig_2.0.3             htmltools_0.4.0
## [35] limma_3.40.6                rlang_0.4.6
## [37] ggthemes_4.2.0              readxl_1.3.1
## [39] TTR_0.23-6                  farver_2.0.3
## [41] generics_0.0.2              zoo_1.8-8
## [43] mclust_5.4.6                BiocParallel_1.18.1
## [45] dplyr_1.0.0                 zip_2.0.4
## [47] car_3.0-8                   RCurl_1.98-1.2
## [49] magrittr_1.5                GenomeInfoDbData_1.2.1
## [51] Matrix_1.2-18               Rcpp_1.0.4.6
## [53] munsell_0.5.0               S4Vectors_0.22.1
## [55] abind_1.4-5                 lifecycle_0.2.0
## [57] scatterplot3d_0.3-41        stringi_1.4.6
## [59] yaml_2.2.1                  carData_3.0-4
## [61] MASS_7.3-53                 SummarizedExperiment_1.14.1
## [63] zlibbioc_1.30.0             grid_3.6.3
## [65] parallel_3.6.3              forcats_0.5.0
## [67] crayon_1.3.4                lattice_0.20-41
## [69] haven_2.3.1                 hms_0.5.3
## [71] pillar_1.4.4                igraph_1.2.5
## [73] ranger_0.12.1               GenomicRanges_1.36.1
## [75] boot_1.3-25                 marray_1.62.0
## [77] stats4_3.6.3                glue_1.4.1
## [79] evaluate_0.14               data.table_1.12.8
## [81] laeken_0.5.1                BiocManager_1.30.10
## [83] vcd_1.4-7                   vctrs_0.3.0
## [85] VIM_6.0.0                   cellranger_1.1.0
## [87] gtable_0.3.0                purrr_0.3.4
## [89] ggplot2_3.3.1               xfun_0.14
## [91] openxlsx_4.1.5              RcppEigen_0.3.3.7.0
## [93] e1071_1.7-3                 qlcMatrix_0.9.7
## [95] class_7.3-17                tibble_3.0.1
## [97] IRanges_2.18.3              ellipsis_0.3.1
```

References

Chu, Li-Fang, Ning Leng, Jue Zhang, Zhonggang Hou, Daniel Mamott, David T. Vereide, Jeea Choi, Christina Kendziorski, Ron Stewart, and Thomson James A. 2016. “Single-cell RNA-seq reveals novel regulators of human embryonic stem cell differentiation to definitive endoderm.” *Genome Biology* 17 (1): 173.

Teschendorff, A. E., and N. Wang. 2020. “Improved Detection of Tumor Suppressor Events in Single-Cell Rna-Seq Data.” *NPJ Genom Med* 5: 43.

Teschendorff, Andrew E, and Tariq Enver. 2017. “Single-cell entropy for accurate estimation of differentiation potency from a cell’s transcriptome.” *Nature Communications* 8 (1): 15599.

Teschendorff, Andrew E, Peter Sollich, and Reimer Kuehn. 2014. “Signalling entropy: A novel network-theoretical framework for systems analysis and interpretation of functional omic data.” *Methods* 67 (3): 282.

Yang, L., W. H. Wang, W. L. Qiu, Z. Guo, E. Bi, and C. R. Xu. 2017. “A Single-Cell Transcriptomic Analysis Reveals Precise Pathways and Regulatory Mechanisms Underlying Hepatoblast Differentiation.” *Hepatology* 66 (5): 1387–1401.
